# Early motor learning changes in upper-limb dynamics and shoulder complex loading during handrim wheelchair propulsion

**DOI:** 10.1186/s12984-015-0017-5

**Published:** 2015-03-10

**Authors:** Riemer J K Vegter, Johanneke Hartog, Sonja de Groot, Claudine J Lamoth, Michel J Bekker, Jan W van der Scheer, Lucas H V van der Woude, Dirkjan H E J Veeger

**Affiliations:** University of Groningen, University Medical Center Groningen, Center for Human Movement Sciences, Groningen, The Netherlands; Amsterdam Rehabilitation Research Center Reade, Amsterdam, The Netherlands; Swiss Paraplegic Research, Nottwil, Switzerland; University of Groningen, University Medical Center Groningen, Center for Rehabilitation, Groningen, The Netherlands; Faculty of Human Movement Sciences, Research Institute MOVE, Vrije Universiteit, Amsterdam, The Netherlands; Faculty of Mechanical, Maritime and Materials Engineering, Section Biomechatronics & Biorobotics, Delft University of Technology, Delft, The Netherlands

**Keywords:** (MeSH), Biomechanics, Motor learning, Rehabilitation, Optimization, Wheeled mobility

## Abstract

**Background:**

To propel in an energy-efficient manner, handrim wheelchair users must learn to control the bimanually applied forces onto the rims, preserving both speed and direction of locomotion. Previous studies have found an increase in mechanical efficiency due to motor learning associated with changes in propulsion technique, but it is unclear in what way the propulsion technique impacts the load on the shoulder complex. The purpose of this study was to evaluate mechanical efficiency, propulsion technique and load on the shoulder complex during the initial stage of motor learning.

**Methods:**

15 naive able-bodied participants received 12-minutes uninstructed wheelchair practice on a motor driven treadmill, consisting of three 4-minute blocks separated by two minutes rest. Practice was performed at a fixed belt speed (v = 1.1 m/s) and constant low-intensity power output (0.2 W/kg). Energy consumption, kinematics and kinetics of propulsion technique were continuously measured. The Delft Shoulder Model was used to calculate net joint moments, muscle activity and glenohumeral reaction force.

**Results:**

With practice mechanical efficiency increased and propulsion technique changed, reflected by a reduced push frequency and increased work per push, performed over a larger contact angle, with more tangentially applied force and reduced power losses before and after each push. Contrary to our expectations, the above mentioned propulsion technique changes were found together with an increased load on the shoulder complex reflected by higher net moments, a higher total muscle power and higher peak and mean glenohumeral reaction forces.

**Conclusions:**

It appears that the early stages of motor learning in handrim wheelchair propulsion are indeed associated with improved technique and efficiency due to optimization of the kinematics and dynamics of the upper extremity. This process goes at the cost of an increased muscular effort and mechanical loading of the shoulder complex. This seems to be associated with an unchanged stable function of the trunk and could be due to the early learning phase where participants still have to learn to effectively use the full movement amplitude available within the wheelchair-user combination. Apparently whole body energy efficiency has priority over mechanical loading in the early stages of learning to propel a handrim wheelchair.

## Background

Persons with a lower-limb disability often depend on a handrim-propelled wheelchair for mobility during daily life. Handrim wheelchair propulsion is a physically straining form of ambulation as a consequence of a low mechanical efficiency and a high mechanical load on the shoulder complex, which might be associated with the frequent over-use injuries of the shoulder in people with a spinal cord injury [1-7].

Different studies on motor learning of wheelchair propulsion have shown that on a group level low-intensity practice can change the propulsion technique of handrim wheelchair propulsion and improve the mechanical efficiency [8-15], which is the ratio of external power output over internal power production. Furthermore, it was found that the propulsion technique changes because of practice, towards a longer-slower movement pattern with an increased angle of hand to rim contact and more net work per cycle, consequently reducing the push frequency [16,17]. However, it is currently not clear in what way these changes in propulsion technique impact the load on the shoulder complex.

To evaluate the load on the shoulder complex during a push cycle, inverse dynamics can be used as input for a musculoskeletal model to estimate muscle activity and joint reaction forces. For experienced wheelchair users the Delft Shoulder and Elbow Model [18] estimated peak glenohumeral reaction forces between 300 to 1400 N during each push cycle at speeds between 0.4 and 1.5 m^.^s^−1^, with concomitant high relative forces of the rotator cuff muscles, especially of the subscapularis and infraspinatus muscles [3,19-21]. When taking into account that wheeling an hour a day with a typical push frequency of 45 pushes per minute may already add up to some 2700 repetitions, the associated load on the shoulder complex might be considered a risk factor for overuse injury of the rotator cuff [22] and shoulder in general. Therefore, it is important to investigate whether motor learning-associated changes in propulsion technique are related to a reduction of the muscle forces and joint reaction forces of the shoulder complex.

In the present study the effect of natural motor learning on propulsion technique, shoulder load and mechanical efficiency will be studied in a group of novice able-bodied participants during the first twelve minutes of low-intensity wheelchair practice. Previously, this relatively short time frame of practice already showed improvements in mechanical efficiency and propulsion technique while at the same time also showing motor learning differences between a group of slow and fast improvers [16,17]. The slow and fast improvers were identified based on a relative 10% increase in mechanical efficiency over a 12 min practice period. The fast learning group increased more in mechanical efficiency and propulsion technique over the whole practice intervention. The current study will enroll a group of able-bodied novices in the same experimental protocol and - by adding three-dimensional position registration - will also be able to use the Delft Shoulder and Elbow Model [18] to evaluate the consequences of three bouts of 4 min low-intensity natural steady state wheeling practice on a motor driven treadmill on mechanical efficiency, propulsion technique, and on the modeled loading of the shoulder complex.

Therefore the objective of the current study was to establish whether the motor learning process during the first 12 minutes of handrim wheelchair propulsion would lead to 1) an increased mechanical efficiency and propulsion technique; 2) a reduction of mean and peak net moments around the glenohumeral shoulder joint and elbow; 3) a reduction of muscle activation and glenohumeral joint reaction force of the shoulder complex; 4) differences in the effect of practice between two groups of learners based on mechanical efficiency and reflected in propulsion technique and load on the shoulder complex.

It is hypothesized that because of practice the participants will change their propulsion technique towards a less straining mode of wheelchair propulsion [16,17], i.e. an increase in mechanical efficiency, adaption of a longer-slower movement pattern and a reduction in muscle forces and consequent glenohumeral reaction forces. In line with the results of our previous study we expected to identify two different groups of learners.

## Methods

### Participants

Fifteen able-bodied novices (8 male, 7 female), with a mean age of 27.4 ± 11.9 years, mean mass of 70.6 ± 13.6 kg and mean height of 1.78 ± 0.09 m, participated in the research after giving informed consent. Criteria for inclusion were: being able-bodied and having no previous experience with wheelchair propulsion. The exclusion criterion was the presence of any severe medical conditions that could have an influence on parameters measured in this study, based on a questionnaire (PAR-Q, ACSM (2009)). The study was approved by the Local Ethics Committee, of the Center for Human Movement Sciences, University Medical Center Groningen, University of Groningen, the Netherlands.

### Protocol

**Figure 1:**
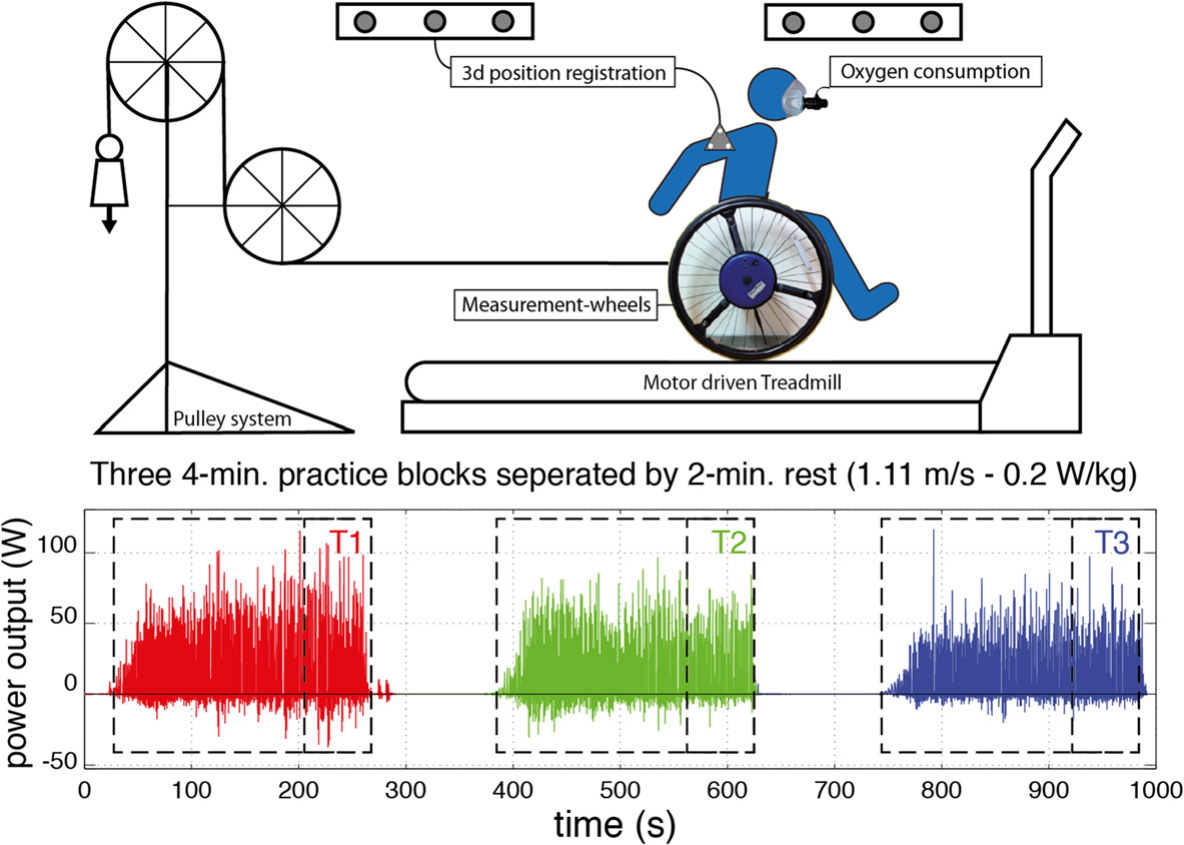
**Schematic overview of the experimental setup and data collection.** Top: Experimental measurement and test set-up for low-intensity steady state (v = 1.1 m/s; 0.2 W/kg body weight) treadmill wheeling, using a pulley system, measurement wheels, mobile oxygen uptake and 3D motion capture on a motor driven treadmill. Bottom: Example of the momentary power output (W) over the whole 12 min practice protocol (3x4min; 2 min rest in between) for one participant. Data of the last minute of each practice block was analyzed (T1, T2, and T3).

The single session 16-minute experiment was conducted on a level treadmill of 2.4 m length by 1.2 m width (Forcelink^a^) in an experimental wheelchair (Double Performance^b^) with 24-inch measurement wheels (Figure 1, top). Each participant performed three consecutive 4-minute exercise blocks at a fixed submaximal power output of 0.20 W/kg body weight with two minutes of rest in between blocks. This low intensity was chosen to minimize fatigue or training effects and focus primarily on motor learning. The first 40 seconds were used to get the treadmill up to a speed of 1.11 m/s (4 km/h). Participants received no specific instructions other than to stay on the treadmill using the handrims. Apart from rolling resistance, the required power output was imposed by adding mass to a pulley system (Figure 1, top). Pulley mass was determined from the results of an individual wheelchair drag test [23,24].

### Energy expenditure

Oxygen consumption (VO2) was continuously measured during each practice session using breath-by-breath open circuit spirometry^e^. The gas analyzer was calibrated using a Jaeger 5 l syringe, room air and a calibration gas mixture. Data collected over the fourth minute of each exercise trial were averaged and taken to reflect physiological steady-state wheelchair propulsion. From the VO2 (L/min), VCO2 (L/min) and respiratory exchange ratio (VCO2/VO2) the energy expenditure was determined using the formula proposed by Garby and Astrup [25]. Mechanical efficiency was derived from the ratio between the external power output (W) and the energetic equivalent of oxygen uptake (W) and (Table 1).

**Table 1:** **Propulsion technique variables and their definitions, automatically processed from the wheel signals using custom written Matlab code** [26]

**Variable**	**Description**	**Equation**
Mechanical efficiency (%)	The percentage of internal power used for external power delivered at the wheels	Mean power output/Energy expenditure
Push time (s)	Time from the start of positive torque to the stop of positive torque for an individual push.	t_end_(i) ‐ t_start_(i)
Cycle time (s)	Time from the start of positive torque to the next start of positive torque.	t_end_(i) ‐ t_start_(i ‐ 1)
Frequency (push*min^−1^)	The number of complete pushes per minute.	N_pushes_/Δt
Work/push (J)	The power integrated over the Contact angle of the push.	∑_start : end_(Tz * ΔØ)
PnegS (W)	The minimum power preceding the push phase	Min_<start_(Power)
PnegE (W)	The minimum power following the push phase	Min_>end_(Power)
Contact angle (°)	Angle at the end of a push minus the angle at the start.	Ø_end_(i) ‐ Ø_start_(i)
Ftot_mean_ (N)	3d mean force within the push phase	Mean_start : end_(Fx^2^ + Fy^2^ + Fz^2^)^0.5^
Ftot_peak_ (N)	3d peak force within the push phase	Max_start : end_(Fx^2^ + Fy^2^ + Fz^2^)^0.5^
FeF_mean_ (%)	Mean Fraction effective Force	Mean_start : end_(F_tangential_/F_total_)
GH start position (mm)	Horizontal position of the glenohumeral joint (GHx) at the start of the push with respect to the wheel-axle (WAx)	GHx_start_(i) ‐ WAx_start_(i)
GH displacement (mm)	The position difference between GH at the start and end of the push phase	GH_end_(i) ‐ GH_start_(i)

*Abbreviations*: *t* time(s), _start_(i), start of the current push (sample); _end_(i), end of the current push (sample); Ø, angle (rad); Fx, Fy and Fz, force components (N); Tz, torque around wheel axle (Nm).

### Measurement wheels

The regular rear wheels of the standardized wheelchair were replaced with one of two instrumented wheels, the Optipush^c^ (Max Mobility) or the Smartwheel^d^ (3-Rivers). Both wheels measure 3-dimensional forces and torques applied to the handrim, combined with the angle under which the wheel is rotated. Data were wirelessly transferred to a laptop at 200 Hz. An electronic pulse at the start of each measurement synchronized both wheels. Data of both wheels show good comparability, with an intra-class correlation for absolute agreement (ICC) of 0.89 for mean power output and ICC’s higher than 0.90 for propulsion technique characteristics [26].

### Propulsion technique

The data from the instrumented wheels were further analyzed using custom-written Matlab routines. To be certain of stable, steady-state wheelchair propulsion, each last minute from the 4-min trials was used for the analysis. Per participant and exercise block the measured force (N), torque (Nm), angle (rad) and time (s) were used for further analyses. Individual pushes were defined as each period of continuous positive torque around the wheel axis with a positive minimum of at least 1 Nm [26]. Over the identified pushes the propulsion technique variables (Table 1) were calculated and subsequently averaged over all pushes within the fourth minute of each practice trial per participant.

### Kinematics

**Figure 2:**
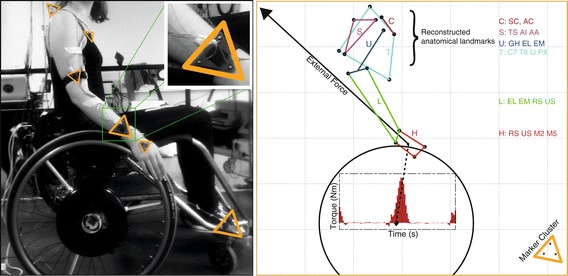
**Example of the marker placements and the reconstruction of anatomical landmarks.** Left: Placement of the technical marker clusters during active wheeling on the motor driven treadmill. Right: Combination of kinematics and wheel kinetics showing a sample of the individual external reaction force and resulting torque around the wheel-axle, during the push phase. The anatomical landmarks used for the thorax (T) clavicle (C) Scapula (S) Upper arm (U) Lower arm (L) and Hand (H) comply with the ISB recommendations [27].

Kinematic data were collected using an optoelectronic camera system (Optotrak, Northern Digital, Waterloo, Canada) at 100Hz with technical cluster markers attached to the right side of the participants’ body and to the wheelchair (Figure 2, left). Prior to the actual experiment, a calibration measurement was performed to determine the location of anatomical landmarks in relation to their technical clusters. From these calibrations, the positions of the anatomical landmarks were reconstructed during the experiment (Figure 2, right), which in turn were used to construct joint coordinate systems of the shoulder, elbow and wrist [27]. The location of the glenohumeral (GH) rotation point was calculated using the regression method proposed by Meskers et al. [28].

### Delft shoulder and elbow model

The Delft Shoulder and Elbow Model (DSEM) is a finite-element, inverse dynamic model describing musculoskeletal behaviour of the upper extremity. Kinematic input was the position of the incisura jugularis, the orientations of the thorax, scapula, humerus, forearm and hand. The 3-dimensional external forces applied by the hand on the handrim served as kinetic input. Five regular consecutive pushes were selected for data analysis. The output of the model is twofold (Table 2). First inverse dynamical calculation takes into account the external forces and accelerations to calculate net moments around the glenohumeral shoulder joint and humeroulnar joint. From this input the model simulates the activity of 31 muscles, divided in 155 elements and the consequent joint reaction forces. The non-individualized anthropometric parameters are based on two cadaver studies [29]. Muscle forces were calculated by an energy related cost function [30]. To enable interpretation and comparison of muscle forces, forces were also expressed as percentages of their maximum based on a force per physiological cross-sectional areas of these muscles of 100 N*cm^−2^, taking into account that the physiological cross-sectional area was measured in an older specimen [29], while the task is performed by young participants.

**Table 2:** **DSEM outcome variables**

**Variable**	**Description**
GH mean Net Moment/Push (Nm)	The mean external net moment of the reaction force around the glenohumeral joint
GH peak Net Moment/Push (Nm)	The peak external net moment of the reaction force around the glenohumeral joint
HU mean Net Moment/Push (Nm)	The mean external net moment of the reaction force around the Humeroulnar joint
HU peak Net Moment/Push (Nm)	The peak external net moment of the reaction force around the Humeroulnar joint
Muscle Power total mean/Push (W)	The mean sum of all muscle powers during the push
Muscle Power total peak/Push (W)	The peak sum of all muscle powers during the push
Muscle Work total/Push (J)	The total muscle work performed per push
GH Reaction force mean/Push (N)	The mean glenohumeral reaction force per push
GH Reaction force peak/Push (N)	The peak glenohumeral reaction force per push
GH Reaction force peak/Cycle (N)	The peak glenohumeral reaction force per cycle

### Statistics

All data were checked for normal distribution and qualified for parametric statistical testing. To evaluate the effect of practice time repeated-measures ANOVA was used to compare mechanical efficiency, propulsion technique parameters, net joint moments of the glenohumeral and humeroulnar joint and the resulting muscular activity and glenohumeral joint reaction forces. Significance for the repeated-measures ANOVA was set at a p < 0.05 and by use of the Bonferroni correction the significance for the post hoc t-tests between any of the three different blocks was set at p < 0.017.

The relationship between the mean net joint moment and the mean glenohumeral joint reaction force was evaluated using a linear least square regression.

To examine motor learning differences between participants, the group was split in two sub-groups, based on a relative increase in mechanical efficiency of more than 10% between T1 and T3, to ensure that differences in learning were above the natural expected variation [17]. The two groups were subsequently compared on the main outcome measures over all three practice-blocks using repeated-measures Anova, with the interaction between group (≤10% or >10%) and practice-blocks as the most important outcome.

## Results

**Figure 3:**
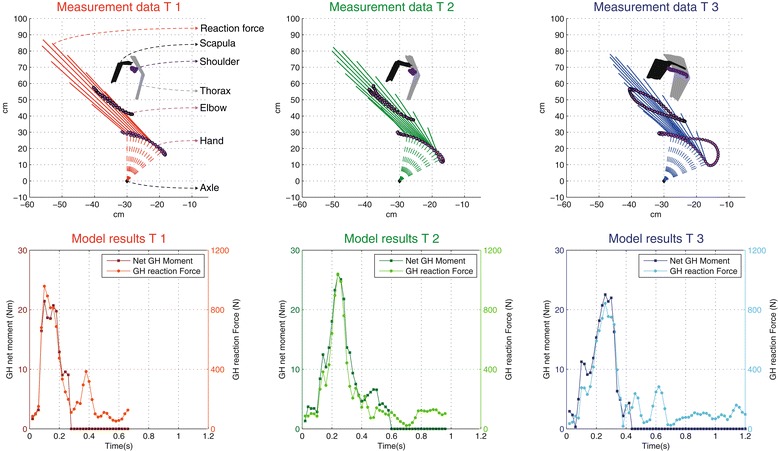
**Typical example of one push cycle for each trial (row 1) and consequent DSEM outcomes (row 2) over the 12 min natural learning period, derived from the input from the T1, T2 and T3 measurements.** The 1^st^ (top) row shows the kinematic and kinetic input (reaction force vector) for the model in relation to the trajectories of the shoulder, elbow and hand over the push and recovery phase (Cycle) at T1, T2 and T3. The 2^nd^ row shows the shoulder loading as expressed in both total net moment (Nm) and total joint reaction force (N) over this same cycle at T1, T2 and T3.

Participants practiced at an average power output of 16.5 ± 3.4 W. Figure 3 shows a typical example of the data collections and outcomes for a push cycle at T1, T2 and T3.

### Effect of motor learning on mechanical efficiency and propulsion technique

The mechanical efficiency significantly increased (T1: 5.5%, T2: 5.9%, T3: 6.0%) over the practice time (Table 3). The post-hoc comparison however only showed a significant difference between T1-T3.

**Table 3:** **Mean (+/− sd) outcomes for all participants (n = 15) over the three consecutive practice blocks and outcomes of statistical analyses (levels of significance: P-Anova: <0.05; Bonferonni tests: <0.017)**

		**T1 Mean Sd**		**T2 Mean Sd**		**T3 Mean Sd**		**p-Anova**	**p-T1T2**	**p-T1T3**	**pT2T3**
	Mechanical Efficiency	**5.5**	1.1	**5.9**	1.1	**6.0**	1.3	**0.047**	0.039	0.014	0.266
Propulsion Technique	Push time (s)	**0.31**	0.06	**0.34**	0.06	**0.34**	0.06	**0.003**	0.006	0.007	0.202
	Cycle time (s)	**0.97**	0.24	**1.15**	0.26	**1.15**	0.24	**0.004**	0.004	0.012	0.471
	Frequency (push/min)	**66.6**	17.5	**55.5**	12.8	**55.0**	11.9	**0.004**	0.007	0.010	0.371
	Work/push (J)	**8.7**	2.1	**10.3**	3.0	**10.3**	3.0	**0.009**	0.009	0.019	0.599
	PnegS (W)	**−6.1**	3.3	**−4.8**	2.3	**−4.3**	2.1	**0.003**	0.011	0.007	0.027
	PnegE (W)	**−3.7**	2.8	**−2.6**	1.9	**1.9**	1.9	**0.012**	0.048	0.011	0.014
	Contact angle (°)	**63.5**	12.1	**69.6**	10.9	**70.4**	10.8	**0.002**	0.005	0.005	0.216
	Ftotmean (N)	41.5	10.3	41.8	14.2	40.4	13.1	0.672	0.877	0.562	0.167
	Ftotpeak (N)	68.0	17.2	69.6	24.1	66.5	22.2	0.553	0.632	0.650	0.082
	FeFmean (%)	**69.4**	10.4	**74.8**	11.6	**75.3**	9.9	**0.000**	0.002	0.001	0.342
	GH start position (mm)	−41	33	−53	35	−47	47	0.247	0.081	0.454	0.388
	GH displacement (mm)	27	13	37	31	39	21	0.245	0.297	0.116	0.770
Net Moments	GH mean Net Moment per Push (Nm)	**12.4**	3.4	**16.1**	5.9	**15.3**	3.4	**0.010**	0.010	0.000	0.700
	GH mean Net Moment per Push (Nm)	26.3	8.1	31.0	12.7	28.5	7.0	0.270	0.080	0.120	0.770
	HU mean Net Moment per Push (Nm)	1.6	2.6	0.9	2.1	0.8	2.7	0.423	0.284	0.309	0.779
	HU peak Net Moment per Push (Nm)	7.9	4.2	6.6	3.2	7.0	3.5	0.452	0.268	0.443	0.636
Model Results	Muscle Power total mean per push (W)	**25.1**	11.0	**35.0**	14.7	**37.5**	15.4	**0.012**	0.008	0.018	0.561
	Muscle Power total peak per push (W)	110.7	49.0	120.5	52.8	134.5	108.6	0.566	0.543	0.222	0.645
	Muscle Work total mean per push (J)	**11.3**	4.0	**15.1**	6.7	**16.1**	7.8	**0.040**	0.014	0.005	0.333
	GH Reaction force mean per push (N)	**315**	107	**419**	171	**439**	165	**0.006**	0.001	0.003	0.337
	GH Reaction force peak per push (N)	690	320	790	390	901	560	0.265	0.119	0.031	0.268
	GH Reaction force peak per cycle (N)	**239**	41	**266**	58	**277**	68	**0.007**	0.006	0.005	0.187

Significant results are presented in bold font style.

For the timing of propulsion technique significant increases in push time (T1: 0.31 s, T2: 0.34 s, T3: 0.34 s) and cycle time (T1: 0.97 s, T2: 1.15 s, T3: 1.15 s) were found with significant post-hoc differences between T1-T2 and T1-T3. The increase in cycle time was also reflected by the reduced push frequency (T1: 66.6, T2: 55.5, T3: 55.0 pushes per minute) with similar significant post-hoc differences between T1-T2 and T1-T3. The positive work per push went up (T1: 8.7 J, T2: 10.3 J T3: 10.3 J), but again showing post-hoc effects only between T1-T2 and T1-T3. The negative phases before the push (T1: −8.1 W, T2: −6.1 W, T3: −5.5 W) and after the push (T1: −5.0 W, T2: −3.9 W, T3: −2.8 W) significantly reduced each next trial.

The increased work per push was performed over a larger contact angle on the handrim (T1: 63.5, T2: 69.6 T3: 70.4 degrees), rather than by an increase of force application. The latter is expressed in the absence of change in both Ftot_mean_ (T1: 41.5 N, T2: 41.8 N, T3: 40.4 N) and Ftot_peak_ (T1: 68.0 N, T2: 69.6 N T3: 66.5 N). The mean fraction effective force showed a significant increase (T1: 69.4%, T2: 75.4%, T3: 75.3%), but again showing post-hoc effects only between T1-T2 and T1-T3.

The start position of the glenohumeral joint in the sagittal plane at the start of the push did not increase significantly over time (T1: −41 mm, T2: −53 mm, T3: −47 mm). Also the following displacement during the push did not increase significantly over time (T1: 27 mm, T2: 37 mm, T3: 39 mm), suggesting a rather inert trunk position during the propulsion cycle.

### Effect of motor learning on shoulder complex loading

The mean net moment of the external force over the push phase around the glenohumeral joint significantly increased (T1: 12.4 Nm, T2: 16.1 Nm, T3: 15.3 Nm) with significant post-hoc differences between T1-T2 and T1-T3. The peak net moment of the external force around the glenohumeral joint did not increase significantly over time (T1: 26.3 Nm, T2: 31.0 Nm, T3: 28.5 Nm). Around the humeroulnar joint no significant changes in mean net moment (T1: 1.6 Nm, T2: 0.9 Nm, T3: 0.8 Nm) or peak net moment (T1: 7.7 Nm, T2: 6.6 Nm, T3: 7.0 Nm) were present over time.

In line with the increased net moments around the glenohumeral joint, the total mean muscle power per push, as estimated from the DSEM, increased significantly (T1: 25.1 W, T2: 35.0 W, T3: 37.5 W), with post-hoc difference seen for T1-T2 and T1-T3. No significant increase in peak power was observed (T1: 110.7 W, T2: 120.5 W, T3: 134.5 W). Also, the total muscle work per push increased over time (T1: 11.3 J, T2: 15.1, T3: 16.1 J), with post-hoc differences for T1-T2 and T1-T3.

A significant increase was found for the mean glenohumeral reaction force per push (T1: 315 N, T2: 419 N, T3: 439 N) with post-hoc differences for T1-T2 and T1-T3. This increase per push also resulted in an increased mean glenohumeral force per cycle (T1: 239 N, T2: 266 N, T3: 277 N), with post-hoc differences again seen between T1-T2 and T1-T3 (Table 3). The peak glenohumeral reaction force did not significantly increase over time (T1: 690 N, T2: 790 N, T3: 901 N).

**Figure 4:**
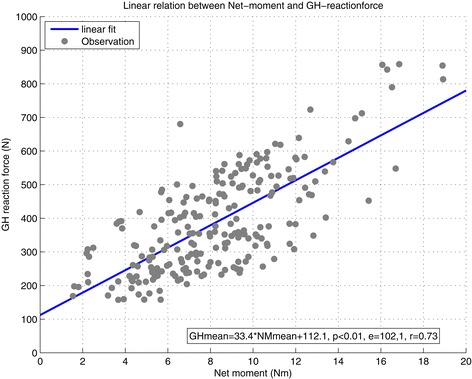
**Linear relationship between the net joint moments (M_dsem) and total compression forces (F_dsem) in the GH joint, shown by the following regression equation:** **F _ dsem = 33.4 ∗ M _ dsem + 112.1** **, with p < 0.01, e = 102.1 N and r = 0.73.**

The increase in net moments and glenohumeral reaction force indicate an increased load on the shoulder complex. Over all observations of all participants a linear relationship was found between the net joint moments (M_dsem) and total compression forces (F_dsem) in the GH joint, shown by the following regression equation: F _ dsem = 33.4 ∗ M _ dsem + 112.1, with p < 0.01, e = 102.1 N and r = 0.73 (Figure 4).

### Moment-balances

**Figure 5:**
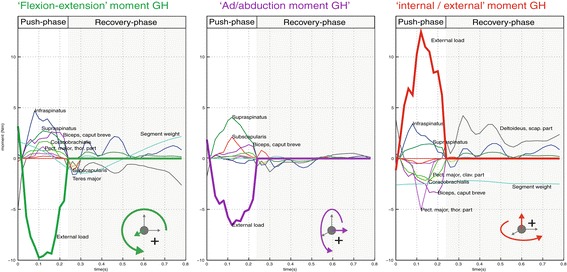
**Example of the muscles- vs. external moment-balance around the Glenohumeral joint for the three global axes, as determined by the DSEM for an individual push at T3.**

Figure 5 shows a typical example of the different muscle contributions that counteract the external moment around the glenohumeral joint for each of the three global axes. Around the global x-axis, mainly the infraspinatus, subscapularis and biceps muscles are responsible for the ‘flexion’ moment, with smaller contributions of the coracobrachialis and pectoralis major. Around the global y-axis the supraspinatus, supscapularis and biceps mostly account for the ‘adduction’ moment. The moment around the global z-axis is mainly expressed by pectoralis major, biceps and coracobrachialis activity, but besides the external moment these muscles also have to counteract the vector components of the infra- and supraspinatus in this plane. The potential consequences of motor learning for this typical pattern over time are described below.

### Effect of motor learning on individual muscle activity

#### Main drivers

**Figure 6:**
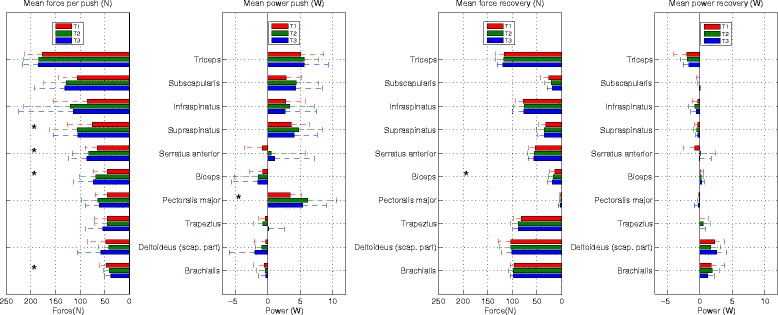
**Outcomes (n = 15; mean +/−sd) of the DSEM for individual muscle forces (N) and powers (W) during the push (left) and recovery (right) phases over time (T1-T3).** Only depicted are those muscles that had mean muscle forces during the push phase larger than 25 N.

The triceps showed the highest mean forces over time (T1: 176 N, T2: 184 N, T3: 185 N), with a large positive contribution to power development around the elbow (T1: 5.1 W, T2: 5.6 W, T3: 5.7 W), but both force and power did not change significantly over time (Figure 6). The highest mean forces leading to positive power development around the shoulder were found in the rotator-cuff muscles subscapularis (T1: 106 N, T2: 129 N, T3: 132 N), infraspinatus (T1: 86 N, T2: 120 N, T3: 114 N), and supraspinatus (T1: 75 N, T2: 106 N, T3: 105 N), of which only supraspinatus expressed a significant change over time at group level (T1-T2 and T1-T3). The mean force of the serratus anterior (T1: 65 N, T2: 83 N, T3: 87 N) increased significantly between T1-T2 and T1-T3. Although this did not lead to a significant change in power output it is noticeable that its mean power contributions are negative at T1 and positive at T3 (T1: −0.9 W, T2: 0.6 W, T3: 1.1 W). The mean force of the biceps (T1: 44 N, T2: 68 N, T3: 74 N) increases significantly between T1-T2 and T1-T3. Figure 5 shows the positive contribution of the biceps to flexion/extension and ad/abduction around the shoulder, but since the biceps is a bi-articular muscle, its force delivers a moment around both shoulder and elbow and a negative power contribution is found (T1: −0.7 W, T2: −1.5 W, T3: −1.5 W), which did not increase over time. Although a trend was present (p < 0.1), the mean force of the pectoralis major (T1: 45 N, T2: 65 N, T3: 61 N) did not increase significantly, but a significant increase of positive power (T1: 3.5 W, T2: 6.2 W, T3: 5.4 W) was found for T1-T2. The mean force of the trapezius (T1: 45 N, T2: 45 N, T3: 54 N) and scapular part of the deltoideus (T1: 49 N, T2: 42 N, T3: 47 N) did not increase over time. The power production of these two muscles was negative and also did not change significantly (T1: −0.4 W, T2: −0.8 W, T3: −0.1 W) and (T1: −0.3 W, T2: −0.9 W, T3: −0.1 W). The muscle force of the brachialis (T1: 47 N, T2: 41 N, T3: 38 N) significantly decreased between T1-T2 and T1-T3, but no significant change was found for the power production (T1: −0.6 W, T2: −0.4 W, T3: −0.3 W).

### Relative muscle activity

**Figure 7:**
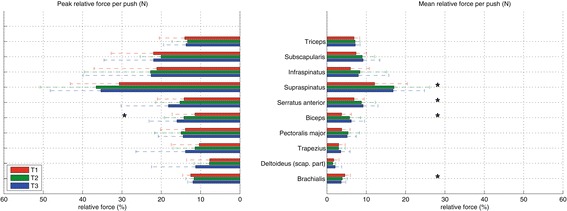
**Relative mean forces (n = 15) of individual muscles during the push phase.** The error bars represent the standard deviation. Only depicted are those muscles that had mean muscle forces during the push phase larger than 25 N.

The contributions of individual muscles relative to their theoretical maximum force (Figure 7) gives a perspective on those muscles that may be at risk for overuse. The supraspinatus is the most taxed muscle during the push phase, of which the mean relative force (T1: 12.1%, T2: 17.1%, T3: 16.8%) significantly increased for T1-T2 and T1-T3, but with no significant increase in the maximum relative force (T1: 30.7%, T2: 36.6%, T3: 35.4%). The biceps was the only muscle to significantly increase in peak relative muscle force (T1: 11.4%, T2: 14.2%, T3: 16.0%), with significant increase between T1-T2 and T1-T3.

### Individual differences in learning

**Figure 8:**
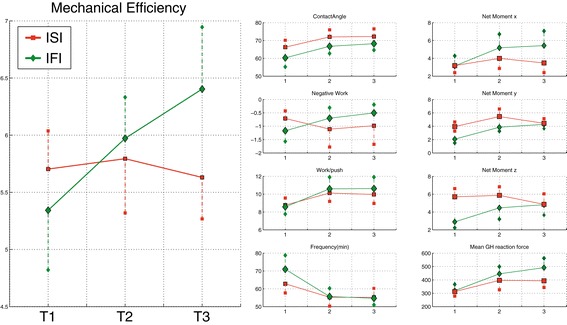
**Differences between initially fast improvers (n = 7; IFI; green) and initially slow improvers (n = 8; ISI, red).** Only mechanical efficiency shows a significant interaction effect between Initially Slow Improvers (ISI) and Initially Fast Improvers (IFI) over practice time (T1-T3).

Seven participants could be classified as Initially Fast Improvers and the other eight as Initially Slow Improvers. A significant interaction was found for mechanical efficiency (Figure 8), but not for the propulsion technique variables or the net moments or the model results.

## Discussion

Because of practice an increase was found in mechanical efficiency over time, indicating that overall less energy was used to maintain a constant speed and power output in the wheelchair on the motor driven treadmill. A concomitant change in propulsion technique was expressed in a reduced push frequency and increased amount of work per push, performed over a larger contact angle with reduced power losses before and after a push, where mean and peak total force in the push remained constant over time. Simultaneously, the fraction effective force increased, indicating a more tangential direction of the applied forces around the wheel-axle. Contrary to our expectations, the above-mentioned propulsion technique changes were found together with an increased net moment, increased total muscle power and increased total muscle work around the glenohumeral shoulder joint. Consequently, this resulted in higher local strains in the shoulder complex as expressed in higher mean and peak glenohumeral reaction forces during both the push-phase as well as the full propulsion-cycle over time.

The current study evaluated the same motor learning process of a steady-state cyclical task on three distinct levels of task execution; the mechanical efficiency encompasses the whole body physiological outcome, the propulsion technique reflects the wheelchair-user interaction at the hand and handrim and the DSEM gives the most detailed description of changes on the level of the shoulder complex. The relations among these three levels are discussed below in the context of the constant experimental conditions and task of maintaining an average power output (0.2 W/kg) and treadmill speed (1.11 m/s) over time; given this common task different relations can be presumed among the different outcomes of these different levels of measurement.

### Effect of motor learning on mechanical efficiency and propulsion technique

The increased mechanical efficiency indicates a more optimal task performance, i.e. energy efficient changes within the body as a consequence of task execution characteristics, among others propulsion technique. The propulsion technique changes that were previously reported to relate most to the increased mechanical efficiency over the initial 12 minutes indeed changed in the current study, i.e. a reduced negative work per cycle, an increased contact angle, an increased work per cycle and consequently a reduced push frequency [17].

In other cyclical tasks the reduced energy cost also coincided with an increase in movement amplitude and a decrease of movement frequency, described as a longer-slower movement pattern [31-35]. Similar to those observations the reduction of the push frequency as a consequence of motor learning is thought to be key to all other propulsion technique changes seen in this cyclic synchronous upper body task [36]: it reduces the repetitiveness of arm motions, which leads to less moments of peak strain and less negative work because of the reduction of the number of (de)coupling of the hands onto the handrim per time unit. An additional increase in movement amplitude and performed work might have been achieved by use of the trunk muscles [37]. However, no increase of trunk motion, i.e. no increase in GH displacement in the sagittal plane, was observed with practice. Possibly, in this early phase of learning the users are still solving the control problem of wheelchair propulsion by maintaining a fairly rigid trunk orientation, instead of already fully using the movement amplitude of the trunk as can be observed in more trained wheelchair-users with adequate trunk control [38].

The Fraction effective Force increased on average 5% between T1 and T3 in the current experiment, which indicates a more tangential orientation of the total force vector of the hand on the rim. This is more than in our previous study on natural learning of handrim propulsion [17], where in a larger group only an increase of 2% was found. As this increase is the consequence of non-instructed natural motor learning, this change in FeF is seen as beneficial because less non-propulsive force needs to be applied.

### Effect of motor learning on shoulder complex loading

Contrary to our expectations, the mean net moment per push of the external force around the glenohumeral joint increased over time, indicating a higher load on the shoulder complex. This implies that the force of the hand on the handrim increased in vector length and/or in moment arm with respect to the glenohumeral joint over time. However, no changes in mean or peak total force of the hand on the handrim were found over time. Therefore, the change in the mean net moment is mainly attributed to changes in moment arm, which is in accordance with the observed increase of the fraction effective force. Another potential factor that might have influenced the moment arm is the position of the glenohumeral joint with respect to the external applied force, but no changes were found in the position or displacement of the glenohumeral joint over time.

Following the same trend as the net moments, the total muscle power and total muscle work around the glenohumeral shoulder joint increased with practice. Given the reduced push frequency, by definition an increased work per push on the wheel is necessary to maintain power output [24]. From our results the increase in total muscle work is larger then the increase in work per push at the wheel. Possibly, for an increase in positive work of the muscles extra work is necessary to stabilize the joint, since the shoulder joint unlike the hip needs more active muscle control for joint stability [39].

The higher estimated muscle activity, as expressed by the increased muscle power and muscle work, resulted in higher mean glenohumeral reaction forces during both the push-phase and the whole push-cycle over time. The average glenohumeral peak force at T3 was around 900 N, which is in accordance with previously reported values [3].

The net moments and the joint reaction force of the glenohumeral joint showed a moderately strong linear relationship. This was previously reported for abduction in static tasks [40] with a fairly similar slope (33.4 vs. 35.3), but with a different intercept (112.1 N vs. 8.12 N). The net glenohumeral joint moment appears to be a good indicator for mechanical load in the glenohumeral joint for the dynamic wheelchair propulsion task.

### Effect of motor learning on individual muscle activity

The activity of the triceps in this group of young able-bodied novices is higher than reported in an EMG study during this initial phase of learning on an ergometer [41] and also higher than reported in more experienced users [3,42]. The triceps as a group have the highest physiological cross sectional area of all muscles and during this initial phase of learning appear to be the prime muscle power producers [43].

The rotator-cuff muscles subscapularis, infraspinatus and supraspinatus, three prime stabilizers of the glenohumeral joint, are highly active during the push phase, especially relative to their limited muscle mass; their activity is comparable to the activity reported by other studies with more experienced users [3,42]. Moreover, because of practice even an increase in the supraspinatus activity is seen that contributes to positive power. The only other muscle that significantly increased in mean force over time and contributed to positive power is the serratus anterior. Even though no significant change in power of the serratus anterior was shown, it is a muscle that at T1 had a mean negative power and at T2 and T3 a mean positive power. The muscle helps to protract the scapula around the thorax and might depending on the timing be able to deliver more positive power.

An increase in biceps and decrease in brachialis activity was observed with practice. Both deliver negative power around the elbow, i.e. increase in muscle length, but the biceps also has an important contribution to counteract the net moment around the shoulder (Figure 5). The negative power contributions of the elbow flexors are in line with the previously stated suggestions for a low mechanical efficiency [44]. Although increased biceps activity might have helped with the more tangential force direction, because the negative power observed in the biceps allows the direction of the external force to come closer to or cross over the elbow, its function can be described as a balance between cost and effect, since the mechanically required and biomechanically preferred force directions are not in accordance with each other [45].

An increase in the power of the pectoralis major was found with practice, with a trend of increased muscle force. Also in other studies the pectoralis major was shown to be one of the major power contributors [42,46,47]. Finally, the contribution of the clavicular part of the deltoideus was very low in these novice wheelchair-users, while previously this was reported to be a main contributor [41,42,46].

### Individual differences in learning

**Figure 9:**
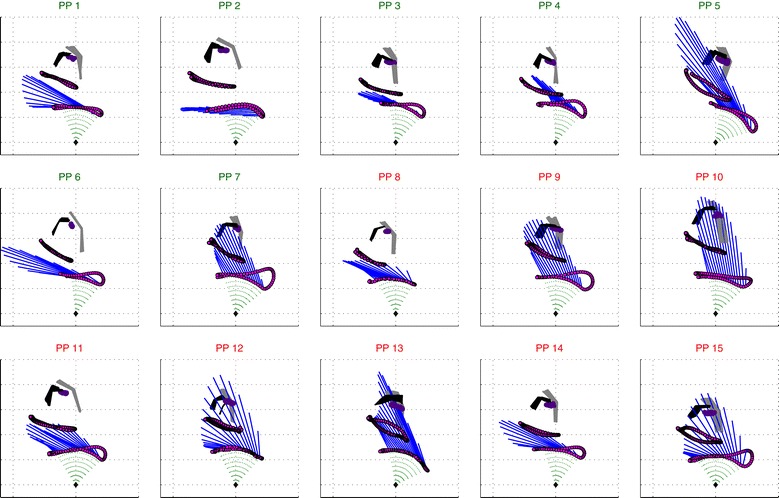
**One push cycle for each individual in the 12** ^**th**^ **minute of practice.** Participants are categorized on the classification of initially fast Improvers (IFI, green title) and initially slow improvers (ISI), red title).

Seven initially fast improvers and eight initially slow improvers were identified; this is relatively more slow learners than found in a larger group of 70 participants with 46 vs. 24 respectively [17]. The curves for mechanical efficiency in the current study look fairly similar compared to a previous study with the slow learners showing a steady line around 5.7% and the fast learners initially starting lower and increasing over time. However, apart from mechanical efficiency, in the current experiment no significant interactions were present in any of the propulsion technique measures or in the load on the shoulder complex. This might be due the high standard deviations in performance outcomes within the limited sample-size of this group. The goal of the experiment was to look at common motor learning changes because of practice at different levels of observation. However, since no individual adjustments were made to the wheelchair every participant was confronted with a slightly different wheelchair-user interface as a consequence of body size vs the constant wheelchair configuration for all participants, while seat-height, chair-width and weight distribution are considered important factors for wheelchair propulsion [48-55]. On a group level an increase in mechanical efficiency showed that individuals were able to optimize within the task constraints, however the optimal solution is suspected to be different based on the constraints-based framework proposed by Sparrow and Newell [56]. Although Figure 9 needs to be interpreted with caution, given the large intra-individual variability it gives a view on the large inter-individual differences still present during the final minute of practice.

### Clinical relevance

Little is known about the upper-body strain of wheelchair propulsion during the initial stages of wheelchair propulsion during rehabilitation, while at the same time shoulder pain is already present at the start of active rehabilitation [57] and at discharge was recently reported as high as 39% of 138 of persons with a newly acquired spinal cord [58]. The inexperienced able-bodied group in the current study showed a high load on the rotator-cuff muscles subscapularis, infraspinatus and supraspinatus, possibly placing them at risk for over-use injury. Novice wheelchair-users during rehabilitation that are still recovering from the recent trauma are expected to be more vulnerable and although the chosen intensity had low impact on the cardio-respiratory system it may cause a high local risk for overuse of the rotator-cuff muscles already in the very first stage of rehabilitation wheelchair practice. Moreover, with practice the load on the shoulder complex increased instead of reduced. Therefore the design of practice interventions aimed at improving propulsion technique and physical capacity should be evaluated on their impact on the shoulder, balancing stress and recovery.

Continued practice over a longer time scale by able-bodied participants [8-17] and by wheelchair-dependent persons [59] has been shown to further improve mechanical efficiency and propulsion technique, however the findings of the current study emphasize the need to further explore the consequences of motor learning and possible physical adaptations for the local strain on the shoulder complex, using a combination of modeling, kinematics and kinetics. In addition to wheelchair practice aimed at improving the skill of wheelchair users, the provision of shoulder strengthening and handcycling exercises might improve the strength as well as the muscle imbalance of the shoulder muscles to possibly protect them from overuse injury [60-62].

### Limitations

The Delft shoulder model does not individualize to the anthropometrics of an individual but translates the measured values onto a cadaver based model. Although the values of the model showed reasonable agreement with EMG and an instrumented shoulder joint [18,63], the absolute values should be taken with caution. Fortunately the entire data recording was done in a single session, so each next trial was performed with the same placement of technical markers and calibrations of the measurement devices. Therefore, the same input was used on the same model to say something about change over time on a group level.

## Conclusion

Over the first 12 minutes of practice naive able-bodied participants increased their mechanical efficiency, indicating that less energy was used to maintain a constant speed and power output. A change in propulsion technique was shown by a reduced push frequency and increased work per push, performed over a larger contact angle with reduced power losses before and after a push and a more tangentially applied force. Contrary to our expectations, the above-mentioned propulsion technique changes were found together with an increased net moment, increased total muscle power and increased total muscle work around the glenohumeral joint. Consequently, this resulted in higher mean and peak glenohumeral reaction forces. This could be due to the early learning phase where participants still have to learn to effectively use the full movement amplitude available within the wheelchair-user combination. Apparently whole body energy efficiency has priority over mechanical loading in early stages of learning to propel a handrim wheelchair.

## Endnotes

^a^Forcelink b.v, Culemborg The Netherlands.

^b^Double Performance BV, Gouda, The Netherlands.

^c^Three Rivers Holdings, Mesa, AZ, USA.

^d^MAX Mobility, LLC, Antioch, TN, USA.

^e^Oxycon Pro-Delta, Jaeger, Hoechberg, Germany.
